# Predictors and Clinical Characteristics of Relapses in LGI1-Antibody Encephalitis

**DOI:** 10.1212/NXI.0000000000200228

**Published:** 2024-04-11

**Authors:** Lucia Campetella, Antonio Farina, Macarena Villagrán-García, Marine Villard, Marie Benaiteau, Noémie Timestit, Alberto Vogrig, Géraldine Picard, Véronique Rogemond, Dimitri Psimaras, Marie Rafiq, Eve Chanson, Cecile Marchal, David Goncalves, Bastien Joubert, Jérôme Honnorat, Sergio Muñiz-Castrillo

**Affiliations:** From the French Reference Center for Paraneoplastic Neurological Syndromes and Autoimmune Encephalitis (L.C., A.F., M.V.-G., M.V., M.B., A.V., G.P., V.R., B.J., J.H., S.M.-C.), Hospices Civils de Lyon; MeLiS - UCBL-CNRS UMR 5284 - INSERM U1314 (L.C., A.F., M.V.-G., M.B., A.V., B.J., J.H., S.M.-C.), Université Claude Bernard Lyon 1, France; Department of Neuroscience (A.F.), Psychology, Pharmacology and Child Health, University of Florence, Italy; Department of Biostatistics (N.T.), Hospices Civils de Lyon, France; Clinical Neurology (A.V.), Santa Maria Della Misericordia University Hospital, Azienda Sanitaria Universitaria Friuli Centrale (ASU FC); Department of Medicine (DAME) (A.V.), University of Udine, Italy; Neurology Department 2-Mazarin (D.P.), Hôpitaux Universitaires La Pitié Salpêtrière-Charles Foix, APHP; Brain and Spinal Cord Institute (D.P.), INSERM U1127/CNRS UMR 7255, Université Pierre-et-Marie-Curie, Universités Sorbonnes, Paris; Neurology Department (M.R.), Hôpital Pierre Paul Riquet, CHU de Toulouse; Neurology Department (E.C.), Centre Hospitalier Universitaire Gabriel Montpied, Clermont-Ferrand; Neurology Department (C.M.), Centre Hospitalier Universitaire de Bordeaux; Immunology Department (D.G.), Hôpital Lyon Sud, Hospices Civils de Lyon, France; and Stanford Center for Sleep Sciences and Medicine (S.M.-C.), Stanford University, Palo Alto, CA.

## Abstract

**Background and Objectives:**

Relapses occur in 15%–25% of patients with leucine-rich glioma-inactivated 1 antibody (LGI1-Ab) autoimmune encephalitis and may cause additional disability. In this study, we clinically characterized the relapses and identified factors predicting their occurrence.

**Methods:**

This is a retrospective chart review of patients with LGI1-Ab encephalitis diagnosed at our center between 2005 and 2022. Relapse was defined as worsening of previous or appearance of new symptoms after at least 3 months of clinical stabilization.

**Results:**

Among 210 patients, 30 (14%) experienced a total of 33 relapses. The median time to first relapse was 23.9 months (range: 4.9–110.1, interquartile range [IQR]: 17.8). The CSF was inflammatory in 11/25 (44%) relapses, while LGI1-Abs were found in the serum in 16/24 (67%) and in the CSF in 12/26 (46%); brain MRI was abnormal in 16/26 (62%) relapses. Compared with the initial episode, relapses manifested less frequently with 3 or more symptoms (4/30 patients, 13% vs 28/30, 93%; *p* < 0.001) and had lower maximal modified Rankin scale (mRS) score (median 3, range: 2–5, IQR: 1 vs 3, range: 2–5, IQR: 0; *p* = 0.001). The median mRS at last follow-up after relapse (2, range: 0–4, IQR: 2) was significantly higher than after the initial episode (1, range: 0–4, IQR: 1; *p* = 0.005). Relapsing patients did not differ in their initial clinical and diagnostic features from 85 patients without relapse. Nevertheless, residual cognitive dysfunction after the initial episode (hazard ratio:13.8, 95% confidence interval [1.5; 129.5]; *p* = 0.022) and no administration of corticosteroids at the initial episode (hazard ratio: 4.8, 95% confidence interval [1.1; 21.1]; *p* = 0.036) were significantly associated with an increased risk of relapse.

**Discussion:**

Relapses may occur years after the initial encephalitis episode and are usually milder but cause additional disability. Corticosteroid treatment reduces the risk of future relapses, while patients with residual cognitive dysfunction after the initial episode have an increased relapse risk.

## Introduction

Leucine-rich glioma-inactivated 1 antibody (LGI1-Ab) autoimmune encephalitis commonly affects older men and typically presents as a limbic encephalitis with predominant memory and behavioral disturbances, accompanied by sleep disorders, temporal lobe seizures, and, in nearly 30% of the patients, pathognomonic faciobrachial dystonic seizures (FBDS).^1,2^ Although most patients experience a monophasic course followed by a slow recovery with frequent (mainly cognitive) sequelae, relapses have been reported in approximately 15%–25% of patients.^3-8^

In clinical practice, discerning between residual symptoms of LGI1-Ab encephalitis and relapses may be challenging. Early recognition and treatment of relapses is crucial because they may cause additional disability and worsen prognosis.^3,9^ Even more importantly, identifying risk factors of relapse would allow for preventive strategies to be implemented early in the disease course to avert relapse occurrence altogether.

Nevertheless, a thorough characterization of the relapses is lacking. Despite a few recent reports attempting to describe them in more detail,^3,4,6,10^ the heterogeneous inclusion criteria and modest sample sizes did not allow for any conclusive inference. Notably, it is currently unclear whether relapsing patients are clinically distinctive and whether particular immunomodulatory treatments reduce the risk of relapse. In this study, we describe and clinically characterize the relapses in a cohort of relapsing patients with LGI1-Ab encephalitis and investigate potential predictors of relapse.

## Methods

We retrospectively reviewed patients with LGI1-Abs diagnosed at the study center between June 2005 and June 2022; detection of LGI1-Abs in the serum and/or CSF was performed using indirect immunofluorescence on rat brain slides and a cell-based assay (CBA) with human embryonic kidney cells (HEK293) overexpressing LGI1 (both in-house), as previously described.^11,12^ Patients were considered LGI1-Ab positive when autoantibodies were detected by both techniques in the CSF and by CBA in the serum. Inclusion criteria were CNS involvement (LGI1-Ab encephalitis) without an alternative diagnosis, absence of co-occurring neural antibodies, and at least 1 relapse. Patients with a short follow-up limited to the initial encephalitis episode (<3 months) or with insufficient clinical data to define the occurrence of a relapse were excluded. Relapse was defined as either a worsening of previous symptoms or appearance of new ones after clinical stabilization causing neurologic deterioration and not due to concurrent medical conditions, lifestyle changes, or abrupt therapeutic modifications. Clinical stabilization was retrospectively defined as the time point after the acute phase of encephalitis when neurologic improvement was no longer observed and clinical status remained unchanged after at least 3 months, with or without neurologic sequelae. The median time from the initial encephalitis onset to relapse was calculated in a preliminary analysis and was approximately 2 years. Subsequently, only patients with LGI1-Ab encephalitis with no relapse after a follow-up ≥ 2 years were selected and included as a comparison group.

Demographic and clinical data collected were age, sex, diagnostic delay, clinical manifestations (classified into 5 major categories: memory, psychiatric/behavioral, seizures, FBDS, and sleep), maximal modified Rankin scale (mRS) score at encephalitis nadir, T2-weighted fluid-attenuated inversion recovery mesiotemporal lobe (MTL) hyperintensities on brain MRI, EEG findings, hyponatremia (serum sodium ≤132 mEq/L), inflammatory CSF (defined as protein content >0.5 g/L and/or white blood cell count >5 cells/µL and/or presence of oligoclonal bands), CSF positivity for LGI1-Abs, human leukocyte antigen (HLA) DRB1*07:01 carrier status, immunotherapy including first-line treatment (corticosteroids, IV immunoglobulin [IVIG], and plasma exchange), second-line treatment (rituximab and cyclophosphamide), and chronic oral immunosuppression (azathioprine and mycophenolate mofetil), delay to treatment, time to clinical stabilization, and clinical outcome at last follow-up (mRS, cognitive sequelae, and epilepsy). Poor outcome was defined as mRS >2. Epilepsy was defined as reappearance of at least 1 unprovoked seizure after clinical stabilization. Cognitive impairment was classified into 7 categories, from 0 to 5, through an in-house scale focused on evaluating the impact of cognitive dysfunction on the ability to perform previous activities of daily living (ADL): 0 = no cognitive impairment; 0.5 = cognitive symptoms, not impairing everyday activities; 1 = mild cognitive deficits, independent but partial return to previous activities; 2 = mild/moderate cognitive deficits, independent but no return to previous activities or requires minor assistance in instrumental ADL (IADL); 3 = severe cognitive deficits, requires major assistance in IADL; 4 = severe cognitive deficits, requires major assistance in ADL; and 5 = dependent in all ADL. In addition, serum LGI1-Ab titration at the initial episode was performed in patients with a serum sample available within 3 months from the encephalitis diagnosis; titers were determined by end point dilutions on CBA.

### Statistical Analysis

Categorical variables are described as numbers and percentages, and continuous variables are reported as median (range, interquartile range [IQR]). Univariate analysis using the Fisher exact test and χ^2^ test for categorical variables and the Mann-Whitney *U* test for continuous variables was performed to compare the relapsing patients with the control group. The McNemar test and Wilcoxon test for paired samples were used to compare the initial encephalitis episode and relapse. To identify potential predictors of relapse, a Cox proportional hazards regression model was built, including variables pertaining to the initial episode that were either clinically relevant for a potential association with relapse and/or had a *p* value <0.2 in the univariate analysis, adjusting for age, sex, diagnostic delay, and delay to treatment. All statistical tests were 2-sided, and a *p* value <0.05 was considered significant. Statistical analysis was performed using GraphPad Prism version 9.4.1 (GraphPad Software, San Diego, CA).

### Standard Protocol Approvals, Registrations, and Patient Consents

The Institutional Review Board of the Université Claude Bernard Lyon 1 and the Hospices Civils de Lyon approved the study (LGI1-IRM 22-5017). Written informed consent was obtained from all patients for the storage and use of biological samples and clinical information for research purposes.

### Data Availability

Anonymized data not published within this article will be made available by request from any qualified investigator.

## Results

Among the 317 patients diagnosed with LGI1-Abs during the study period, 107 (34%) were excluded due to short follow-up or insufficient clinical information (85/107, 79%), concomitant positivity for other neural antibodies (13/107, 12%), isolated peripheral nervous system involvement (7/107, 7%), and diagnosis of Alzheimer disease concomitant with encephalitis diagnosis (2/101, 2%). Among the remaining 210 patients, there were 30 (14%) who experienced at least 1 relapse and were included in this study (eFigure 1), for a total of 33 relapses.

The median age at onset of LGI1-Ab encephalitis was 66 years (range: 29–86, IQR: 17), and 23/30 (77%) patients were male. Symptoms at the first episode of encephalitis were memory impairment (30/30, 100%), psychiatric/behavioral symptoms (26/30, 87%), FBDS (14/30, 47%), seizures (24/30, 80%), and sleep disturbances (13/30, 43%). All patients (30/30, 100%) received first-line treatment, and 17/30 (57%) received second-line treatment. Patients reached clinical stabilization in a median 11 months (range: 1.5–25, IQR: 7.2); the median mRS at stabilization was 1 (range: 0–4, IQR: 1; Figure 1). A total of 6/30 (20%) patients had a poor outcome (mRS > 2) at stabilization; 4 of them experienced severe memory impairment and 2 mild cognitive symptoms. Mild/moderate cognitive sequelae were reported in 17 of the remaining 24 (71%) patients with good outcome (Figure 2).

**Figure 1:**
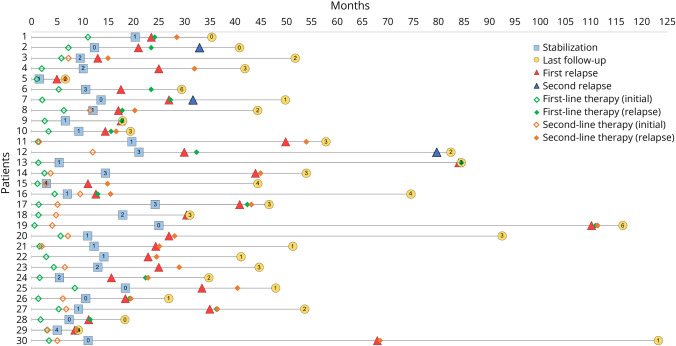
Disease Course and Treatment of Patients With Relapsing LGI1-Ab Encephalitis Swimmer plot illustrating the disease course of the 30 relapsing patients, with time on the abscissa axis extending from clinical onset (at time 0) until the last follow-up (yellow circle). Each patient is represented by a different number and line on the ordinate axis (1–30). The treatment received at the initial episode (green and orange empty rhombi) and the occurrence of a first (red triangle) and second relapse (blue triangle) are illustrated. Rhombi (green and orange) represent the treatment received for the relapse. The time point of clinical stabilization (light blue square) was established retrospectively to mark the beginning of an interval of at least 3 months during which the patient did not manifest any neurologic change. The modified Rankin scale (mRS) scores at clinical stabilization and last follow-up are depicted inside the respective indicators. LGI1-Ab = leucine-rich glioma-inactivated 1 antibody.

**Figure 2:**
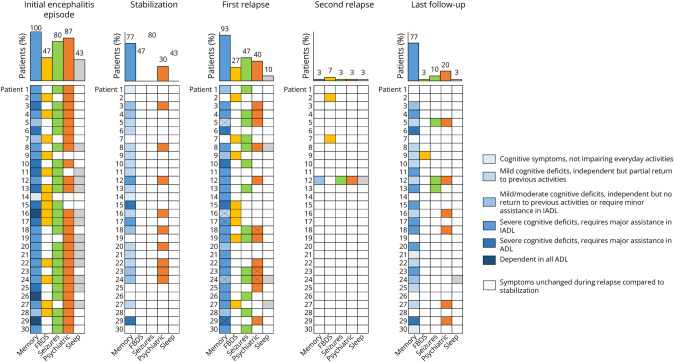
Evolution of Symptoms Before and After the Relapse Heatmap and bar graphs illustrating the prevalence of different symptoms (memory impairment, FBDS, seizures, psychiatric/behavioral symptoms and sleep disturbances) during the initial encephalitis episode, stabilization, first and second relapses, and at last follow-up. The severity of cognitive symptoms is graded through different shades of blue, as specified in the legend. Symptoms that remained stable between stabilization and relapse are marked with a cross. ADL = activities of daily living; FBDS = faciobrachial dystonic seizures; IADL = instrumental activities of daily living; memory = memory impairment; psychiatric = psychiatric/behavioral symptoms; sleep = sleep disturbances.

### Relapse Characteristics

The median time from the LGI1-Ab encephalitis onset to the first relapse was 23.9 months (range: 4.9–110.1, IQR: 17.8; Table 1, Figure 1); 18/33 (55%) relapses occurred more than 2 years after onset. Two patients received LGI1-Ab encephalitis diagnosis during the relapse, when LGI1-Abs were detected in both the serum and CSF. During the initial episode, 1 patient tested negative for LGI1-Abs in the CSF (the serum was not tested), and the other one tested negative twice by indirect immunofluorescence in the serum and CSF; both were consequently diagnosed during the first episode as having “autoimmune encephalitis without antibodies” and received immunotherapy. Eight relapses (24%) occurred while the patients were undergoing immunotherapy: 4 patients with rituximab, 1 with rituximab and corticosteroids, 2 patients with cyclophosphamide and corticosteroids, and 1 with oral corticosteroids only. Among the 5 patients relapsing while on rituximab, CD19^+^ lymphocyte count was 0% and “markedly reduced” in 2 and was not available in the remaining 3 (eTable 1). All the patients who relapsed while on corticosteroids were undergoing slow tapering.

**Table 1:** Summary of Main Clinical and Diagnostic Features During the Relapses

	Relapses, n = 33
Median age at relapse, y (range; IQR)	68 (30–90; 14)
Median time from initial encephalitis onset to first relapse, mo (range; IQR)	23.9 (4.9–110.1; 17.8)
Median time from clinical stabilization to relapse, mo (range; IQR)	10.2 (3–85; 11.2)
Clinical features, n (%)	
Memory impairment	23 (70)
FBDS	10 (30)
Seizures	15 (45)
Psychiatric/behavioral	10 (30)
Sleep disturbances	4 (12)
Median maximal mRS score (range; IQR)	3 (2–5; 1)
Diagnostic findings, n (%)	
Hyponatremia	4/28 (14)
Inflammatory CSF	11/25 (44)
LGI1-Abs in serum	16/24 (67)
LGI1-Abs in CSF	12/26 (46)
T2/FLAIR MTL hyperintensities on brain MRI^a^	14/26 (54)
EEG documenting ictal activity^b^	3/23 (13)
Altered metabolism in brain FDG-PET	2/5 (40)
Treatment^c^, n (%)	
IVIG	9 (27)
Corticosteroids	15 (45)
Rituximab	19 (58)
Cyclophosphamide	11 (33)
Median delay to treatment, d (range; IQR)	38 (3–218; 53.5)
Outcome (n = 30 patients)	
Median time to stabilization, mo (range; IQR)	10 (2.2–20; 10.3)
Median mRS (range; IQR)	2 (0–6; 2)
Poor outcome (mRS >2), n (%)	14 (47)
Cognitive dysfunction, n (%)	23 (77)
Epilepsy, n (%)	2 (7)

Abbreviations: FBDS = faciobrachial dystonic seizures; FDG-PET = fluorodeoxyglucose PET; IQR = interquartile range; IVIG = IV immunoglobulin; LGI1-Abs = leucine-rich glioma-inactivated 1 antibodies; mRS = modified Rankin scale; MTL = mesiotemporal lobe; T2/FLAIR = T2-weighted fluid-attenuated inversion recovery.

a 2/26 patients developed T2/FLAIR hyperintensity of other areas (1 basal ganglia, 1 cerebellar peduncles).

b In the remaining 20 patients, EEG revealed focal or diffuse slowing in 6/23 (26%) and normal findings in 14/23 (61%).

c 9/33 (27%) only first-line treatment (4 corticosteroids, 3 corticosteroids + IVIG, 2 IVIG); 13/33 (39%) only second-line treatment (5 rituximab + cyclophosphamide, 5 rituximab, 3 cyclophosphamide); 10/33 (30%) combination of both (3 corticosteroids + rituximab, 2 IVIG + rituximab, 2 corticosteroids + IVIG + rituximab, 2 corticosteroids + rituximab + cyclophosphamide, 1 corticosteroids + cyclophosphamide).

The most common clinical manifestation during relapse was memory impairment (23/33, 70%), followed by seizures (15/33, 45%), FBDS (10/33, 30%; isolated in 5/33, 15%), and psychiatric/behavioral symptoms (10/33, 30%; Table 1, Figure 2). Four patients (12%) developed sleep disturbances (1inversion of circadian rhythm, 1 insomnia and vivid dreams, 1 insomnia and limb movements, and 1 hypnopompic hallucinations), while 5/33 (15%) experienced other symptoms: gait instability (2/5), impaired consciousness with abnormal limb movements (1/5), oral dyskinesia (1/5), and aphasia (1/5).

Hyponatremia was observed in 4/28 (14%) relapses. A lumbar puncture was performed during 26 relapses, and the CSF was inflammatory in 11/25 (44%); all 10 patients concerned had previous CSF analysis and 4/10 (40%) had inflammatory CSF at the initial episode. No patient had intrathecal synthesis of oligoclonal bands. LGI1-Abs were positive in the serum of 15/23 (65%) patients and in the CSF of 12/25 (48%; eFigure 2). Given that 1 patient who experienced 2 relapses had serum and CSF samples available both at first and second relapses, LGI1-Abs were overall positive in the serum in 16/24 (67%) relapses and in the CSF in 12/26 (46%) relapses. Notably, LGI1-Abs reappeared after a previous negativity in 4/16 (25%) relapses, while, conversely, LGI1-Abs were not detected in the serum nor the CSF in 7/24 (29%) relapses. A brain MRI was performed in 26 relapses and was abnormal in 16 (62%), documenting stability of previous MTL hyperintensities in 11/26 (42%) cases and novel MTL findings in 3/26 (12%). An EEG was performed in 25 relapses; the final report was available in 23 patients, revealing ictal activity in 3/23 (13%). Neurodegenerative biomarkers (total tau, phosphorylated tau, Aβ42, Aβ40, and ratio Aβ42/Aβ40) were investigated in the CSF of 7 patients, 4 of them having at least 1 abnormal value (data not shown).

Regarding immunotherapy, 9/33 (27%) relapses were treated exclusively with first-line treatment, 13/33 (39%) only with a second-line treatment, and 10/33 (30%) with a combination of both. Overall, IVIG were administered in 9/33 (27%) relapses, corticosteroids in 15/33 (45%), rituximab in 19/33 (58%), and cyclophosphamide in 11/33 (33%; Table 1). One patient (3%) did not receive any specific treatment, although she was lost to follow-up 1 month after the relapse.

The median follow-up after relapse was 15.7 months (range: 0.3–65.6, IQR: 20.8). Ten patients (33%) had not reached clinical stabilization at the last follow-up; of these, 5 were lost to follow-up, 3 had a recent relapse diagnosis (in 2022), and 2 died (one due to SARS-CoV-2–related pneumonia, and cause was unknown in the other). At the last follow-up, the median mRS was 2 (range: 0–6, IQR: 2); 14/30 (47%) patients experienced a poor outcome. Twenty-three patients (77%) presented cognitive sequelae at the last follow-up, of whom 12/23 (52%) previously had residual cognitive dysfunction after the initial episode. One patient was diagnosed with Alzheimer disease (confirmed by CSF biomarkers) more than 1 year after the relapse and had an mRS of 3 at his last visit.

### Comparison Between the Initial Episode and Relapse

We next compared the initial encephalitis episode with the relapse, finding that all symptoms but FBDS were significantly more frequent during the initial event. Overall, 28/30 (93%) patients developed 3 or more symptoms during the initial episode, compared with 4/30 (13%) during relapse (*p* < 0.001). The median maximal mRS was higher during the initial episode than at relapse (median 3, range: 2–5, IQR: 1 vs 3, range: 2–5, IQR: 0; mean ± SD 3.6 ± 0.9 vs 3 ± 0.7; *p* = 0.001).

Regarding diagnostic findings, hyponatremia (4/26, 15% vs 22/30, 73%; *p* < 0.001) and LGI1-Abs in the CSF (12/25, 48% vs 23/29, 79%; *p* = 0.039) were significantly less frequent during the relapse. Relapses were less commonly treated with first-line treatment, and the median delay to immunotherapy was significantly shorter (36.5 days, range: 3–218, IQR: 74.5 vs 81 days, range: 17–360, IQR: 118; *p* = 0.015). Focusing on the 20 patients who reached postrelapse clinical stabilization, the median mRS after the initial episode was 1 (range: 0–4, IQR: 1), while residual disability significantly worsened after the relapse, with a median mRS of 2 (range: 0–4, IQR: 2; *p* = 0.005; Table 2).

**Table 2:** Clinical Features, Treatment, and Outcome of the Initial Episode Compared With First Relapse in the 30 Relapsing Patients

	Initial episode, n = 30	First relapse, n = 30	*p* Value
Clinical features, n (%)			
Memory impairment	30 (100)	23 (77)	0.016
FBDS	14 (47)	8 (27)	0.70
Seizures	24 (80)	14 (47)	0.013
Psychiatric/behavioral	26 (87)	9 (30)	<0.001
Sleep disturbances	13 (43)	3 (10)	0.002
Combination of ≥3 symptoms	28 (93)	4 (13)	<0.001
Median maximal mRS score (range; IQR)	3 (2–5; 1)	3 (2–5; 0)	0.001
Diagnostic findings, n (%)			
Hyponatremia	22 (73)	4/26 (15)	<0.001
Inflammatory CSF	11 (37)	10/24 (42)	0.75
LGI1-Abs in CSF	23/29 (79)	12/25 (48)	0.039
T2/FLAIR MTL hyperintensities on MRI	20/29 (69)	13/25 (52)	0.23
First-line treatment, n (%)	30 (100)	17 (57)	<0.001
IVIG	27 (90)	9 (30)	<0.001
Corticosteroids	25 (83)	13 (43)	0.004
Second-line treatment, n (%)	17 (57)	22 (73)	0.18
Rituximab	14 (47)	18 (60)	0.39
Cyclophosphamide	11 (37)	10 (33)	1
Combination of first-line and second-line treatment	17 (57)	10 (33)	0.09
Median delay to treatment, d (range; IQR)^a^	81 (17–360; 118)	36.5 (3–218; 74.5)	0.015
Outcome			
Median time to stabilization, mo (range; IQR)	11 (1.5–25; 7.2)	12.3 (2.2–20; 10.6)	0.75
Median mRS (range; IQR)^b^	1 (0–4; 1)	2 (0–4; 2)	0.005
Poor outcome (mRS >2)^b^, n (%)	6 (20)	7/20 (35)	0.06
Cognitive dysfunction^b^, n (%)	23 (77)	14/20 (70)	1

Abbreviations: FBDS = faciobrachial dystonic seizures; IQR = interquartile range; IVIG = IV immunoglobulin; LGI1-Abs = leucine-rich glioma-inactivated 1 antibodies; mRS = modified Rankin scale; MTL = mesiotemporal lobe; T2/FLAIR = T2-weighted fluid-attenuated inversion recovery.

a Delay to first-line treatment during the initial episode compared with delay to any treatment during relapse.

b At clinical stabilization after the initial episode vs at clinical stabilization after the relapse (excluding the 10/30 patients who had not stabilized at the last follow-up).

### Comparison of the Initial Episode With the Control Group

To identify potential factors predicting relapse occurrence, we compared the initial episode of patients with relapses with that of 85 nonrelapsing patients. There was no significant difference between the 2 groups regarding main demographical, clinical, and diagnostic findings. Relapsing patients had a shorter delay to first-line treatment (median 81 days, range: 17–360, IQR: 118 vs 145 days, range: 6–750, IQR: 156.5; *p* = 0.013), accompanied by a trend toward earlier second-line treatment and an earlier diagnosis. After the initial episode, there was no significant difference between the 2 groups regarding degree of disability, although the relapsing group had a slightly higher frequency of cognitive sequelae (23/30, 77% vs 51/85, 60%; *p* = 0.1; Table 3). In addition, serum LGI1-Ab titers at the initial episode were determined in 17 relapsing patients and 42 controls with a serum sample available within 3 months from encephalitis diagnosis. There was no significant difference in serum LGI1-Ab titers between the 2 groups, both in the univariate analysis (eTable 2) and in a Cox proportional hazards regression model, correcting for age, sex, diagnostic delay, delay to treatment, and immunotherapy administration (data not shown).

**Table 3:** Characteristics of the Initial Encephalitis Episode in Relapsing Patients Compared With the Control Group

	Relapsing patients, n = 30	Control group, n = 85	*p* Value
Median age at onset, y (range; IQR)	66 (29–86; 17)	64 (21–83; 14)	0.30
Male sex, n (%)	23 (77)	51 (60)	0.10
HLA DRB1*07:01, n (%)	17/22 (77)	53/61 (87)	0.29
Median diagnostic delay, d (range; IQR)	73 (18–3,339; 130)	136 (2–1,530; 161.5)	0.09
Median follow-up, mo (range; IQR)^a^	23.9 (4.9–110.1; 17.8)	36.2 (24.4–156.7; 26)	<0.001
Clinical features, n (%)			
Memory impairment	30 (100)	83 (98)	1
FBDS	14 (47)	45 (53)	0.55
Seizures	24 (80)	73 (86)	0.45
Psychiatric/behavioral	26 (87)	62 (73)	0.13
Sleep disorders	13 (43)	38 (45)	0.90
Combination of ≥3 symptoms	28 (93)	72 (85)	0.35
Median maximal mRS score (range; IQR)	3 (2–5; 1)	3 (1–5; 1)	0.13
Diagnostic findings, n (%)			
Inflammatory CSF	11 (37)	33/82 (40)	0.73
LGI1-Abs in CSF	23/29 (79)	62/79 (78)	0.93
T2/FLAIR MTL hyperintensities on MRI	20/29 (69)	62/84 (74)	0.61
First-line treatment, n (%)	30 (100)	82 (96)	0.57
IVIG	27 (90)	73 (86)	0.76
Corticosteroids (IVMP and/or oral)	25 (83)	67 (79)	0.60
IVMP only	8 (27)	28 (33)	0.52
Median delay onset-start of any first-line treatment, d (range; IQR)	81 (17–360; 118)	145 (6–750; 156.5)	0.013
Second-line treatment, n (%)	17 (57)	48 (56)	0.98
Rituximab	14 (47)	40 (47)	0.97
Cyclophosphamide	11 (37)	34 (40)	0.75
Median delay onset-start of any second-line treatment, d (range; IQR)	167.5 (43–960; 105)	230 (55–1,516; 214.5)	0.09
Chronic oral immunosuppression, n (%)	5 (17)	16 (19)	0.79
Outcome			
Median mRS (range; IQR)^b^	1 (0–4; 1)	1 (0–5; 2)	0.75
Poor outcome (mRS >2)^b^, n (%)	6 (20)	14 (16)	0.66
Cognitive dysfunction^b^, n (%)	23 (77)	51 (60)	0.10
Epilepsy^c^, n (%)	2 (7)	12/79 (15)	0.34

Abbreviations: FBDS = faciobrachial dystonic seizures; HLA = human leukocyte antigen; IQR = interquartile range; IVIG = IV immunoglobulin; IVMP = IV methylprednisolone; LGI1-Abs = leucine-rich glioma-inactivated 1 antibodies; mRS = modified Rankin scale; MTL = mesiotemporal lobe; T2/FLAIR = T2-weighted fluid-attenuated inversion recovery.

a Time to relapse in the relapsing group.

b At clinical stabilization after the initial episode in the relapsing group and at last follow-up in the control group.

c At last follow-up in both the relapsing and control groups.

**Figure 3:**
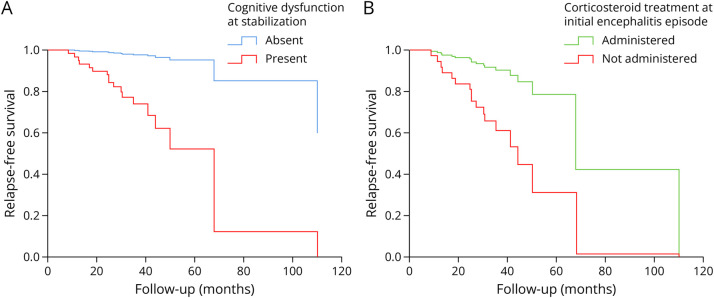
Cox Proportional Hazards Regression Curve for Relapse Occurrence in Patients With and Without Residual Cognitive Dysfunction After the Initial Episode and in Patients With and Without Prior Administration of Corticosteroids at Initial Episode The Cox regression model was built including variables associated with relapse risk. The abscissa axis represents follow-up time (in months) after disease onset. Relapse-free survival on the ordinate axis describes the probability of remaining relapse-free over time. Patients with residual cognitive dysfunction after the initial episode (in red, A) and without prior administration of corticosteroids at initial episode (in red, B) have a higher risk of relapse.

Furthermore, we performed a Cox regression model including the variables age, sex, diagnostic delay, psychiatric/behavioral symptoms, cognitive dysfunction at stabilization, inflammatory CSF, maximal mRS, no administration of corticosteroids, and delay to treatment. The persistence of cognitive dysfunction after the initial episode (hazard ratio: 13.8, 95% confidence interval [1.5; 129.5]; *p* = 0.022) and no administration of corticosteroids at the initial episode (hazard ratio: 4.8, 95% confidence interval [1.1; 21.1]; *p* = 0.036) were significantly associated with an increased risk of relapse (Figure 3, eTable 3, eFigure 3). Although the length of corticosteroid treatment was not available, we performed separate analyses for patients who received IV methylprednisolone (IVMP) alone compared with those treated with IVMP and/or oral corticosteroids and subsequent oral de-escalation. The different Cox regression models built revealed no significant association with relapse risk (data not shown).

In addition, because a substantial number of relapsing patients were treated late in the course of the disease (13/30, 43%), we identified those in whom first-line treatment was administered within the first 3 months, which better reflects the current strategy of early therapeutic intervention, and analyzed factors predicting relapses in this subgroup. We found 17/30 (57%) relapsing patients and 24/85 (28%) controls, for a total of 41 patients, but there was no statistically significant difference regarding their main demographical and clinical features (eTable 4).

## Discussion

Despite being frequently mentioned in the literature, relapses in LGI1-Ab encephalitis have not been systematically described. In this study, we report a series of 30 relapsing patients, providing a detailed outline of the relapses and a comparison with a control group. We found that 14% of patients experience a relapse, sometimes years after the initial episode. Although relapses usually present with fewer and milder symptoms, making them challenging to diagnose, they also lead to a poorer outcome with additional disability. Remarkably, administration of corticosteroids at the initial episode decreases relapse risk, whereas residual cognitive dysfunction after the initial encephalitis episode is a risk factor of relapse.

The frequency of relapses in LGI1-Ab encephalitis varies greatly in the literature, from 13%^4^ to 35%,^2^ and even up to 59% in a mixed cohort including patients with contactin-associated protein-like 2 antibodies.^1^ On one hand, relapses have been heterogeneously defined in the past, likely leading to a misperception regarding their true frequency^1,4,6,8-10^; on the other hand, patients with autoimmune encephalitis may experience transient fluctuations of symptoms early in the disease course,^13^ often due to suspension or dose decrease of immunosuppressive treatment. For instance, FBDS are particularly sensitive to corticosteroids, and fast tapering can prompt their recurrence.^14,15^ Thus, these early fluctuations likely represent an incomplete recovery from the initial episode, principally due to an inappropriate therapeutic management, rather than a true relapse. In this study, we used a more rigorous definition of relapse, similar to a previous work,^8^ to discriminate true relapses from early fluctuations.

In the study population, the median time from onset to relapse was nearly 2 years, with 1 patient relapsing 9 years after the initial episode. Conversely, such a long interval was rare in previous studies, with most patients experiencing a relapse within the first year after onset,^1,6,9^ but this was likely influenced by the aforementioned heterogeneous definitions of relapse. However, in studies with longer follow-up^2^ or clearer distinctions between early fluctuations and relapses,^7^ the interval increases, reaching a median of 35^2^ and 30 months,^7^ which is closer to that found in this study. Hence, even patients showing improvement and clinical stabilization should undergo a long-term neurologic follow-up after the initial episode.

Similar to what has been described in N-methyl-d-aspartate receptor antibody (NMDAR-Ab) encephalitis,^16-18^ relapses in patients with LGI1-Ab do not encompass the typical full-blown syndrome, but present instead with fewer and milder symptoms. As a result, relapses may be subtle and difficult to recognize, especially when only isolated symptoms are present. Furthermore, the distinction between symptomatic seizures in the context of a relapse and autoimmune encephalitis–associated epilepsy^19^ must be carefully evaluated because few patients with LGI1-Ab encephalitis develop epilepsy in the long-term.^8,20^ In addition, infections and changes in lifestyle or medications can worsen cognition in older predisposed patients. Moreover, because the median age of patients with LGI1-Ab encephalitis is relatively high, the possible development of neurodegenerative disorders must be considered, as observed in 1 patient in this study. Thus, when a relapse is suspected, medical history should focus on identifying potential triggers for clinical worsening, and a tailored clinical and diagnostic assessment should be performed to exclude alternative diagnosis.

Laboratory tests were conducted in most relapses described in this study and often found fewer abnormalities compared with the initial episode. Similarly, MRI findings were usually stable when compared with prior examinations, as previously reported.^6^ Furthermore, LGI1-Abs only sporadically resurfaced during relapse after a previous disappearance and were notably absent in both the serum and CSF in almost one-third of patients. This last finding could be explained by a lack of sensitivity of the current antibody detection techniques for low antibody titers. However, few published studies with limited sample sizes report serial antibody testing in LGI1-Ab encephalitis^2,4,9^; one of them found serum LGI1-Abs almost 2 years after treatment in 4 nonrelapsing patients in a cohort of 19, regardless of the outcome.^9^ Hence, the clinical significance of antibody detection during follow-up needs further clarification. Therefore, the diagnosis of a relapse should rely primarily on clinical grounds, although there is certainly an unmet need for objective measures to use as biomarkers of relapse. Because of the retrospective design of this study, we had limited data on the frequency of novel MTL hyperintensities and abnormal neurodegenerative biomarkers in the CSF of relapsing patients. Thus, we could not assess their significance as potential diagnostic and/or prognostic biomarkers of relapse, which should be addressed by future studies.

Of interest, the clinical and diagnostic features at the initial episode were similar between relapsing patients and the control group. Likewise, in recent studies where an analogous analysis was performed, the only significant differences were the frequency of sleep disorders^4^ and residual epilepsy,^3^ both higher in relapsing patients. While LGI1-Ab titers at the initial episode were previously suggested to be associated with relapse occurrence,^10^ we could not reproduce these findings in a subset of patients from this study with available samples. Nonetheless, we observed in this study that persisting cognitive dysfunction after the initial episode was a risk factor of relapse. This is of major importance because a significant number of patients with LGI1-Ab encephalitis report cognitive sequelae,^9,12,21^ although the underlying causes are still incompletely understood, perhaps involving hippocampal atrophy,^22,23^ disrupted connectivity,^24,25^ or reduced cognitive reserve.^26^ Thus, given the elevated disability and the higher risk of relapses, patients with cognitive sequelae should be closely monitored.

Typically, most patients with LGI1-Ab encephalitis have a good prognosis as measured by the mRS,^1,2,9,12,21^ but in this study, a third of the patients who stabilized after the relapse had a poor outcome at the last follow-up. Moreover, median mRS scores were significantly higher after the relapse than after the initial episode, implying that some patients do not return to their prerelapse state but develop additional disability. Similarly, worse cognitive outcome^9^ and incomplete recovery^3^ have previously been associated with relapse in patients with LGI1-Ab encephalitis, corroborating the findings in this study. Thus, preventing relapse occurrence would be of the utmost importance to avoid additional accumulation of disability and poorer outcome.

The role of treatment in relapse prevention has not been comprehensively explored. Lack of immunotherapy, less aggressive treatment regimens, and longer delay to treatment led to a higher risk of relapse in NMDAR-Ab encephalitis.^16,17,27-29^ Rituximab has specifically been suggested to prevent relapses both in patients with NMDAR-Ab encephalitis^30^ and in a mixed cohort of patients with autoimmune encephalitis, including 26 with LGI1-Abs.^31^ Long-term steroid-sparing immunotherapy also reduced relapse risk in a LGI1-Ab encephalitis cohort from a recent study,^8^ although this was not confirmed in a smaller cohort.^32^ In this study, we found that administration of corticosteroids at the initial episode prevents future relapses. The efficacy of corticosteroids in treating manifestations of LGI1-Ab encephalitis such as FBDS^14,15^ and seizures^7,8^ is well-known, and early treatment can prevent the development of cognitive impairment.^33^ Moreover, corticosteroids lead to a greater improvement in disability in the short-term.^7^ Nonetheless, beneficial effects on long-term outcome are less clear,^7^ and chronic administration often leads to adverse events, with significant economic burden on health care systems^34^ and reduced quality of life.^35^ In this study, there was no difference between the impact on relapse risk of either IVMP only or IVMP and/or oral corticosteroids followed by oral tapering. Thus, we cannot currently draw any conclusions about the required duration of the corticosteroid treatment; however, identifying new steroid-sparing drugs with fewer side effects but similar efficacy on relapse prevention remains a clinically imperative objective.

Surprisingly, the delay to treatment was significantly shorter in patients who relapsed than in those who did not. There was also a trend toward a shorter delay to diagnosis, which could seemingly explain the aforementioned finding. We do not have a definite explanation for these shorter delays because there was no significant difference between the 2 groups when compared for the number of symptoms in the initial episode or for clinical severity, as measured through the mRS. However, this could have been influenced by the well-known limitations of the mRS as a measure of disease burden^12,21^ and by the small sample size. Hence, larger cohorts are needed to fully understand the potential differences between relapsing and nonrelapsing patients.

In this study, we provide a detailed clinical and diagnostic picture of the relapses, but the inherent pathogenic mechanisms remain unclear. Given the increased risk in patients with cognitive sequelae, we could hypothesize that a subclinical inflammatory process persists in the temporal lobe, which is preferentially involved in LGI1-Ab encephalitis.^11^ Alternatively, we could speculate that the initial encephalitis event triggers a neurodegenerative process in a subset of predisposed patients and that, over time, progressive neurodegeneration and exhaustion of cognitive reserve allow for new cognitive and behavioral symptoms to manifest, leading to a clinical relapse; moreover, this hypothesis would also explain the ineffectiveness of immunotherapy to prevent relapses. However, other yet unknown genetic and immunologic factors may concur, although we found no association with HLA-DRB1*07:01 carrier status in this study.

Limitations of this study include its retrospective nature, with the consequent lack of longitudinal assessment of diagnostic tests during follow-up and a possible underestimation of subtle and mild symptoms. Second, we cannot exclude that some patients in the control group might have experienced a relapse over a longer follow-up period; however, we specifically selected patients with at least a 2-year follow-up because this interval was consistent with the median time to first relapse among relapsing patients included in this study. Another limitation is the relatively modest sample size; nonetheless, given that LGI1-Ab encephalitis is a rare disease, the present cohort of relapsing patients considerably adds to the available literature. Last, this study showed that radiologic and laboratory inflammatory features, including LGI1-Abs, may be absent in some relapses. However, we recognize the need for objective biomarkers that differentiate more clearly between relapses and clinical fluctuations related to immunotherapy modifications or co-occurring neurodegenerative processes. Nevertheless, the rigorous definition of relapse used in this study and the clinical characteristics of the relapses strongly suggest the autoimmune nature of these events.

In conclusion, relapses may occur years after the initial encephalitis episode and are usually milder but cause additional disability. Of importance, corticosteroid treatment reduces the risk of future relapses, while patients with residual cognitive dysfunction after the initial episode have an increased relapse risk.

## Appendix Authors

**Table:**

Name	Location	Contribution
Lucia Campetella, MD	French Reference Center for Paraneoplastic Neurological Syndromes and Autoimmune Encephalitis, Hospices Civils de Lyon; MeLiS - UCBL-CNRS UMR 5284 - INSERM U1314, Université Claude Bernard Lyon 1, France	Drafting/revision of the article for content, including medical writing for content; major role in the acquisition of data; study concept or design; and analysis or interpretation of data
Antonio Farina, MD	French Reference Center for Paraneoplastic Neurological Syndromes and Autoimmune Encephalitis, Hospices Civils de Lyon; MeLiS - UCBL-CNRS UMR 5284 - INSERM U1314, Université Claude Bernard Lyon 1, France; Department of Neuroscience, Psychology, Pharmacology and Child Health, University of Florence, Italy	Drafting/revision of the article for content, including medical writing for content; analysis or interpretation of data
Macarena Villagrán-García, MD	French Reference Center for Paraneoplastic Neurological Syndromes and Autoimmune Encephalitis, Hospices Civils de Lyon; MeLiS - UCBL-CNRS UMR 5284 - INSERM U1314, Université Claude Bernard Lyon 1, France	Drafting/revision of the article for content, including medical writing for content; analysis or interpretation of data
Marine Villard, MSc	French Reference Center for Paraneoplastic Neurological Syndromes and Autoimmune Encephalitis, Hospices Civils de Lyon, France	Major role in the acquisition of data
Marie Benaiteau, MD	French Reference Center for Paraneoplastic Neurological Syndromes and Autoimmune Encephalitis, Hospices Civils de Lyon; MeLiS - UCBL-CNRS UMR 5284 - INSERM U1314, Université Claude Bernard Lyon 1, France	Drafting/revision of the article for content, including medical writing for content; major role in the acquisition of data; and analysis or interpretation of data
Noémie Timestit, MSc	Department of Biostatistics, Hospices Civils de Lyon, France	Analysis or interpretation of data
Alberto Vogrig, MD, PhD	French Reference Center for Paraneoplastic Neurological Syndromes and Autoimmune Encephalitis, Hospices Civils de Lyon; MeLiS - UCBL-CNRS UMR 5284 - INSERM U1314, Université Claude Bernard Lyon 1, France; Clinical Neurology, Santa Maria Della Misericordia University Hospital, Azienda Sanitaria Universitaria Friuli Centrale (ASU FC); Department of Medicine (DAME), University of Udine, Italy	Drafting/revision of the article for content, including medical writing for content; analysis or interpretation of data
Géraldine Picard, MSc	French Reference Center for Paraneoplastic Neurological Syndromes and Autoimmune Encephalitis, Hospices Civils de Lyon, France	Drafting/revision of the article for content, including medical writing for content; analysis or interpretation of data
Véronique Rogemond, PhD	French Reference Center for Paraneoplastic Neurological Syndromes and Autoimmune Encephalitis, Hospices Civils de Lyon, France	Drafting/revision of the article for content, including medical writing for content; analysis or interpretation of data
Dimitri Psimaras, MD	Neurology Department 2-Mazarin, Hôpitaux Universitaires La Pitié Salpêtrière-Charles Foix, APHP; Brain and Spinal Cord Institute, INSERM U1127/CNRS UMR 7255, Université Pierre-et-Marie-Curie, Universités Sorbonnes, Paris, France	Drafting/revision of the article for content, including medical writing for content; and analysis or interpretation of data
Marie Rafiq, MD	Neurology Department, Hôpital Pierre Paul Riquet, CHU de Toulouse, France	Drafting/revision of the article for content, including medical writing for content; analysis or interpretation of data
Eve Chanson, MD	Neurology Department, Centre Hospitalier Universitaire Gabriel Montpied, Clermont-Ferrand, France	Drafting/revision of the article for content, including medical writing for content; analysis or interpretation of data
Cecile Marchal, MD	Neurology Department, Centre Hospitalier Universitaire de Bordeaux, France	Drafting/revision of the article for content, including medical writing for content; analysis or interpretation of data
David Goncalves, PharmD	Immunology Department, Hôpital Lyon Sud, Hospices Civils de Lyon, France	Drafting/revision of the article for content, including medical writing for content; analysis or interpretation of data
Bastien Joubert, MD, PhD	French Reference Center for Paraneoplastic Neurological Syndromes and Autoimmune Encephalitis, Hospices Civils de Lyon; MeLiS - UCBL-CNRS UMR 5284 - INSERM U1314, Université Claude Bernard Lyon 1, France	Drafting/revision of the article for content, including medical writing for content; analysis or interpretation of data
Jérôme Honnorat, MD, PhD	French Reference Center for Paraneoplastic Neurological Syndromes and Autoimmune Encephalitis, Hospices Civils de Lyon; MeLiS - UCBL-CNRS UMR 5284 - INSERM U1314, Université Claude Bernard Lyon 1, France	Drafting/revision of the article for content, including medical writing for content; study concept or design; and analysis or interpretation of data
Sergio Muñiz-Castrillo, MD, PhD	French Reference Center for Paraneoplastic Neurological Syndromes and Autoimmune Encephalitis, Hospices Civils de Lyon; MeLiS - UCBL-CNRS UMR 5284 - INSERM U1314, Université Claude Bernard Lyon 1, France; Stanford Center for Sleep Sciences and Medicine, Stanford University, Palo Alto, CA	Drafting/revision of the article for content, including medical writing for content; study concept or design; and analysis or interpretation of data

## Glossary

ADL: activities of daily living
CBA: cell-based assay
FBDS: faciobrachial dystonic seizures
HLA: human leukocyte antigen
IQR: interquartile range
IVIG: IV immunoglobulin
IVMP: IV methylprednisolone
LGI1-Ab: leucine-rich glioma-inactivated 1 antibody
mRS: modified Rankin scale
MTL: mesiotemporal lobe
NMDAR-Ab: N-methyl-d-aspartate receptor antibody
